# Prevalence and Causes of Avoidable Blindness in Subjects Over 50 Years of Age in Honduras

**DOI:** 10.18502/jovr.v17i2.10794

**Published:** 2022-04-29

**Authors:** Mariel Eunice Amador Rosa, Alejandra Lozano Bustillo, Iván Espinoza Salvadó, Manuel Sierra, Belinda Rivera

**Affiliations:** ^1^Department of Ophthalmology, Faculty of Medical Sciences, National Autonomous University of Honduras (UNAH); ^2^Ophthalmology Specialist in San Felipe General Hospital

**Keywords:** Blindness, Cataract, Honduras, Prevalence

## Abstract

**Purpose:**

To describe the prevalence and causes of avoidable blindness in people aged 50 and over in the areas of influence of doctors in social service during the years 2018–2019.

**Methods:**

This observational, descriptive, cross-sectional study, with analysis of association of variables, was conducted on patients 50 years and older at the national level, selected under simple random sampling, where sociodemographic variables, background, and clinical characteristics were studied. An ophthalmological clinical examination was performed with prior informed consent, and the information was processed and analyzed using Epi Info 7.2 statistical package and SPSS version 25.

**Results:**

Overall, 7992 people were evaluated, with a mean age of 62 years; 60.8% (4861) were women and 39.2% (3131) were men. The prevalence of blindness for both eyes was 4.5% (356/7992, 95% CI: 4.1–5.1%, *p* < 0.001). The prevalence of severe and moderate visual impairment was 1.5% (118/127) and 12.9% (1029)/12.6% (1004) for the right and left eyes, respectively. The main causes of blindness were cataract, refractive error, and glaucoma.

**Conclusion:**

The prevalence of avoidable blindness found in the study was higher than expected and the respective causes were consistent with previous studies. Consequently, it is recommended to implement health policies aimed at the prevention and management of avoidable blindness.

##  INTRODUCTION

In Latin America and the Caribbean, blindness and vision loss in adults continues to be a substantial public health problem. This challenge that occurs in the 50 years and older demographic is quite impactful on society.^[1]^ According to the World Health Organization (WHO), there are 253 million people with visual impairment, 36 million with blindness, and 217 million with moderate to severe visual impairment. ${}^{\left[2,3\right]}$

The estimated number of blind people increased to 36.0 million in 2015. This change was attributable to three factors, namely an increase due to population growth (38.4%), increase in aging population (34.6%), and reduction in age-specific prevalence (36.7%).^[4,5,6]^ In Honduras, a single study of prevalence of blindness was carried out in 2013 by Alvarado et al. Based on the data from that study, an estimated prevalence of blindness of was 1.9%; the data obtained revealed the magnitude of this serious public health problem.^[7]^ Globally, the causes of both low vision and blindness were uncorrected refractive error, cataracts, age-related macular degeneration, glaucoma, and diabetic retinopathy.^[3,8,9]^ Among some regions of the world, causes of blindness and visual impairment in people over 50 years old varied markedly, with a high prevalence of age-related macular degeneration (>14% blindness) as the primary cause in high-income countries. However, the number of people with visual impairment due to diabetic retinopathy is increasing worldwide and represents an extensive proportion of all causes of blindness.^[9,10,11]^

The prevalence of blindness and severe and moderate visual impairment are concentrated in the most socially disadvantaged sectors of the population.^[12]^ Consequentially in the midst of financial constraints, these communities require the development of eye care systems, including human resources and infrastructure elements to urgently determine the extent of diseases of the anterior and posterior segments of the eye as causes of visual impairment. In this study, we aim to establish the prevalence and causes of avoidable blindness in the 50 years and over segment of the population in the areas of influence which were attended by doctors who provided social services to the communities during the period of April 2018–April 2019. This study's purpose is an attempt to generate interest for the creation of public policies geared toward early diagnosis and timely management of the main causes of blindness.

##  METHODS

This observational, descriptive, cross-sectional study was conducted for a period of one; the first stage consisted of training in basic ophthalmic examination of the social service doctors (last-year students) of the National Autonomous University of Honduras (UNAH) provided by ophthalmology residents and the scientific research unit; the second stage was the evaluation of patients over 50 years by these students; and the third and the final stage was the evaluation and treatment of patients in a specialized ophthalmological consultation in referral centers. For this evaluation, the ophthalmology residents were mobilized to referral centers in different cities of the country.

The training of the last-year students consisted of 5-hr workshops with theoretical modules and supervised practices on visual acuity testing and basic ophthalmological evaluation. Each student was given either a chart with an E optotype or the use of the application "Peek acuity” establishing 6 m as a distance for assessing visual acuity. The protocol was approved by the coordination of the ophthalmology postgraduate, Ethics Committee on Biomedical Research, and Scientific Research Unit of the Medical Sciences Faculty. In our study, we used the classification as:

• Mild: visual acuity worse than 0.3 to 0.5 of LogMAR chart or 20/25 to 20/60 in Snellen chart (SC)
• Moderate: visual acuity worse than 0.5 to 1 or 20/60 to 20/200 in SC
• Severe: visual acuity worse than 1 to 2 or 20/200 to 20/400 in SC
• Blindness: visual acuity worse than 2 or 20/400 in SC

The universe of the study was all nationwide adult patients over 50 years old who lived within the areas of influence by the last-year students who provided the social service. The sampling unit corresponded of a simple random selection of houses in these areas. Prior to the selection, an updated sketch of all the areas of influence was prepared to select the geographic area where the surveys were applied also randomly.

Between November and December 2018, a simple random sample of patients aged 50 years or older from the areas of influence served by the last-year students were selected. A total of 7992 patients were evaluated. Only the subjects that gave their informed consent and fulfilled the inclusion criteria (over 50 years of age and agreed to participate in the study) were considered. Subject with mental disabilities that made impossible to measure visual acuity were excluded from the study.

The students applied a survey that included sociodemographic variables, test of visual acuity (with and without glasses if the patient had it already and also with and without pinhole), personal pathology history, ophthalmological and surgical history, as well as ocular pathological family history and the existence of comorbidities (diabetes mellitus and high blood pressure).In diabetic patients, the onset of the disease and the current treatment were considered. Every student practiced a basic ophthalmological examination and the visual acuity (with and without pinhole) through either printed Snellen primers or the “peek acuity” application which has been used in multiple studies around the world.^[13]^

All patients with visual acuity ≤ 20/200 were sent to reference centers nationwide (Olancho, San Pedro Sula, La Ceiba, Santa Rosa de Copán, San Lorenzo, Comayagua, and Tegucigalpa cities) according to their location where the ophthalmology resident was mobilized to assess them. The cause of blindness or severe visual impairment was then determined and the respective treatment started.

Data were entered into a database created with the statistical package EPIINFO Version 7.2 (CDC, Atlanta USA). The analysis was made with the statistical program SPSS version 25 and consisted of simple frequency construction, univariate and bivariate analysis establishing statistical significance with Chi-square tests for proportion comparison. ORs were constructed with 95% confidence intervals. Statistical significance was considered when "*p*-value" was < 0.05.

##  RESULTS

Of the total of 7992 participants, 3131 (39.2%) were men and 4861 (60.8%) women. Of them, 2042 (25.5%) participants were from the capital and 5950 (74.5%) from the rest of the country. All the study variables are presented in Tables 1–5.

**Table 1:** Characterization of visual acuity per eye in patients aged 50 years and over, Honduras 2018–2019 (*N*: 7992)


**Category**	**Right eye**		**Left eye**	
	***N***	**%**	***N***	**%**
Blindness	220	2.8	234	2.9
Severe visual impairment	118	1.5	127	1.5
Moderate visual impairment	1029	12.9	1004	12.6
Normal/Mild visual impairment	6625	82.9	6627	82.9
	

**Table 2:** Characterization of visual acuity for both eyes in patients aged 50 years and over, Honduras 2018–2019 (*N*: 7992)


**Category**	***N***	**%**
Severe visual impairment	207	(2.5)
Moderate visual impairment	1482	(18.5)
Normal/Mild visual impairment	7114	(89)
	
${}^{*}$ Patients with at least one eye with any of the categories of visual acuity were taken into account		

**Table 3:** Comparison of sociodemographic data of adults aged 50 years and over, with and without blindness in both eyes in Honduras, 2018–2019 (*N*: 7992)


**Characteristic**	**With blindness ** ***N*** **: 356**		**Without blindness ** ***N*** **: 7636**	
Age (yr)	*N*	(%)	*N*	(%)
50–59	70	(19.7)	3799	(49.8)
60–69	116	(32.6)	2179	(28.5)
70–79	93	(26.1)	1196	(15.7)
80–89	70	(19.7)	422	(5.5)
90–105	7	(2)	40	(0.5)
**Sex**		
Female	213	(59.8)	4648	(60.8)
Male	143	(40.1)	2988	(39.1)
**Scholarship**		
Illiterate	123	(34.6)	1519	(19.9)
Primary	191	(53.7)	3882	(50.8)
High school	27	(7.6)	1597	(20.9)
University	15	(4.2)	638	(8.4)
	

**Table 4:** Main causes of blindness in patients aged 50 years or older evaluated in the reference centers, Honduras, 2018–2019 (*N*: 67)


**Cause of blindness**	**Right eye**		**Left eye**		**Both eyes**	
	***N*** **: 67**	**%**	***N*** **: 67**	**%**	***N*** **: 134**	**%**
Cataract without treatment	25	37.3	31	46.3	56	41.7
Refractive error	13	19.4	9	13.4	22	16.4
Glaucoma	8	11.9	9	13.4	17	12.6
Does not apply	2	3.0	0	0	2	1.4
No examination	6	9.0	7	10.4	13	9.7
Another posterior segment	5	7.5	4	6.0	9	6.7
Phthisis	1	1.5		1	0.7
Diabetic retinopathy	6	9.0	4	6.0	10	7.4
Surgical complications	0	0	1	1.5	1	0.7
Other corneal opacities	1	1.5	2	1.5	3	2.2
	
	
${}^{*}$ Patients with blindness in at least one eye were considered						

**Table 5:** Reason why adult patients 50 years or older had not undergone surgery considering themselves blind from cataract, Honduras, 2018–2019 (*N*: 210/206)


**Reason**	**Right eye ** ***N*** **: 210**		**Left eye ** ***N*** **: 206**	
	***N***	**%**	***N***	**%**
No need	37	(17.6)	41	(19.9)
Fear of surgery or poor results	53	(25.2)	55	(26.6)
Can't pay the price	84	(40.0)	87	(42.2)
They refuse to provide the service	8	(3.8)	7	(3.3)
I didn't know there was a treatment	12	(5.7)	11	(5.3)
Cannot travel to care centers	30	(14.2)	27	(13.1)
	
	

##  DISCUSSION

According to global data, of the 7.33 billion people alive by 2015, 36.0 million were blind, 56% of these were women (20.1 million). Most of the world population with blindness is situated in the Asian region.^[14]^ In Latin America, the general prevalence of blindness in people over 50 years varies from region to region.^[1]^ According to the results of our study, 4.5% of people over 50 years of age presented blindness in at least one eye, similar to that described by the WHO for worldwide estimates at 4.24%.^[2]^ Our study also coincides with data from other locations such as three developing world regions in sub-Saharan West Africa, East sub-Saharan Africa, and South Asia with an estimated prevalence of 4%^[14]^ and in countries such as Panama where the prevalence is around 30.0%. It was higher than the result of the only previous study carried out in the country which was 1.9% and studies performed in other countries such as Ecuador (1.7%) and urban areas in Argentina (1.3%), Brazil (1.6%), and Chile (1.4%). The general prevalence of visual impairment (severe and moderate) varies from 8.0% in Uruguay to 14.3% in El Salvador.^[1]^ In our study in Honduras, this group had a percentage of 2.5% with severe visual impairment and 18.5% with moderate visual impairment. Normal vision or mild visual impairment was present in 79%. All this similitude can be related and comparable among the Latin-American region due to factors such as our race, customs and similar challenges as it pertains to access to public health.

There was a higher prevalence of blindness in males but with no statistically significant difference. Our results were different from a comparative study in 2015 on avoidable blindness and visual impairment completed in seven Latin-American countries (Argentina, El Salvador, Honduras, Panama, Paraguay, Peru, Uruguay) where the female population was shown to be predominant in all regions.^[1]^ Globally, the female population is the predominant sex to experience blindness in most of the studies.^[14]^ Another important consideration regarding our study is that most of the surveyed households were answered by the housewives since at that time of the survey, the partner was at work.

Functional presbyopia's prevalence is increasing and is estimated to affect 666.7 million people 50 years of age and over worldwide^[14]^ in the future. In our study, 18.9% patients reported having a history of presbyopia and 26% reported the use of near-vision lenses, which contrasts with global data where the percentage of presbyopia has reached 40%.^[12,14]^

Uncorrected refractive errors in adults have been shown to lead to blindness in some regions.^[15]^ Presbyopia and myopia were the most frequent refractive errors found in our study with 18.9% and 18.3%, respectively. According to the current global data, of the 95 million people over 50 with visual impairment due to uncorrected refractive errors, the presbyopia was the main cause of visual impairment while 6.9 million are blind.

Education status is an important influential social factor in experiencing blindness. In our study, the illiterate patients and those who had attended only primary school accounted for 29% and 53%, respectively, and were the population with a higher index of blindness, similar to that reported by Ko et al who reported that the factors associated with some degree of deterioration, non-refractive visuals included aging, poverty, and a low educational level.^[16]^

From 1990 to 2010, the number of people who were blinded due to cataracts decreased from 12.3 million to 10.8 million.^[17]^ In this study, the personal history recorded of patients revealed that cataract and pterygium surgery were the most prevalent. In countries where the prevalence of cataract is very high, important underlying causes are cataract-induced myopia, uncorrected aphakia, and insufficient lens correction.^[15]^

In this study, the age range of the patients with the highest prevalence of blindness in both eyes was 60–69 years, which differs from world statistics where the highest prevalence is found in a younger population group between the ages of 40 and 49 years.^[17]^

Globally, in 2010, it was determined that cataract causes blidness in a total of 32.4 million people and visual impairments in 191 million.^[17]^ In a Latin-American study, cataract was the main cause of bilateral blindness ranging from 47% to 87%, followed by posterior segment disease, including glaucoma, diabetic retinopathy, and age-related macular degeneration, ranging from 7% to 44%. In our study, cataract was found to be the main cause of blindness at 41.7%, followed by refractive errors at 16.4% and glaucoma and retinopathy with equal percentages of 12.6% and 7.4%, respectively. This data differs from global estimates, where the main causes of visual impairment are reported as uncorrected refractive error and cataracts, 43% and 33%, respectively. ${}^{\left[2,3\right]}$ In countries like Nepal, retinal disorders which account for 46.66% followed by cataract at 43.33% are the two main causes of blindness.^[8]^ Cataract was more common in rural poor areas while retinal disorders were prevalent among the urban population. Of all blindness causes, 88% and 94% were curable and preventable.^[18,19]^

This data differs from global estimates, where the main causes of visual impairment are reported as uncorrected refractive error and cataracts, 43% and 33%, respectively.^[2,3]^ In Bhaktapur district, a popular city of Nepal, retinal disorders which account for 46.66% followed by cataract at 43.33% are the two main causes of blindness.^[8]^

The reason most of the patients in the study experienced blindness due to cataract was as a result of their inability to pay for the surgery. In another study conducted in sub-Saharan Africa, similar causes were discovered that led to patients experiencing blindness because of a treatable illness. The additional barriers to treatment that were discovered included the surgical cost, the lack of family support, the lack of understanding of the need for surgery, and other social, infrastructural, and geographical factors, such as disparity in the distribution of ophthalmologists throughout the region.^[21]^ The quality of cataract surgery also remains a concern, as poor results have reached up to 40% in some areas.^[22]^

In conclusion, the prevalence of blindness in people over 50 years old at the national level in Honduras has increased in the last five years (1.9% to 4.5%) and seems to be closely associated with data from national socioeconomic indicators. Cataract continues to be the most important cause of blindness and it seems that this problem has not changed in the last five years years, despite being previously identified as the major cause of visual impairment and blindness. Underestimating the impact of diabetic retinopathy, glaucoma, or other diseases is easy when there is insufficient data, but they are important causes of blindness that should always be considered.

Socioeconomic factors must be taken into account when implementing strategies to improve the prevention of blindness. Cataract continues to be a challenge to address with the need to plan a comprehensive strategy related to the accessibility of eye care services. Improving the outcome of cataract surgery in the low-income population should also be focused. These initiatives are already conditions outlined to achieve the basic global goal established by the WHO, which accounts for a 25% reduction in the prevalence of avoidable blindness and visual impairment by the year of 2020. The reduction of blindness and visual impairment may be achieved by establishing improvements in the quality of life and advocating for higher incomes for the patient, family, and society to increase accessibility to proper healthcare.

##  Financial Support and Sponsorship

None.

##  Conflicts of Interest

The authors declare no competing interests.
